# The Pathogenesis of Ischemia-Reperfusion Induced Acute Kidney Injury Depends on Renal Neutrophil Recruitment Whereas Sepsis-Induced AKI Does Not

**DOI:** 10.3389/fimmu.2022.843782

**Published:** 2022-04-21

**Authors:** Zhenhan Li, Nadine Ludwig, Katharina Thomas, Sina Mersmann, Martin Lehmann, Dietmar Vestweber, Jean-Francois Pittet, Hernando Gomez, John A. Kellum, Jan Rossaint, Alexander Zarbock

**Affiliations:** ^1^ Department of Anesthesiology, Intensive Care and Pain Medicine, University Hospital Münster, Münster, Germany; ^2^ Department of Vascular Cell Biology, Max Planck Institute for Molecular Biomedicine, Münster, Germany; ^3^ Department of Anesthesiology and Perioperative Medicine, University of Alabama at Birmingham, Birmingham, AL, United States; ^4^ The Center for Critical Care Nephrology, Department of Critical Care Medicine, University of Pittsburgh, Pittsburgh, PA, United States

**Keywords:** neutrophils, kidney, ischemia-reperfusion injury, cecal ligation and puncture, sepsis, acute kidney injury

## Abstract

Acute kidney injury (AKI) may be induced by different causes, including renal ischemia-reperfusion injury and sepsis, which represent the most common reasons for AKI in hospitalized patients. AKI is defined by reduced urine production and/or increased plasma creatinine. However, this definition does not address the molecular mechanisms of different AKI entities, and uncertainties remain regarding distinct pathophysiological events causing kidney injury in the first place. In particular, sepsis-induced AKI is considered not to be associated with leukocyte infiltration into the kidney, but a direct investigation of this process is missing to this date. In this study, we used two murine AKI models induced by either renal ischemia-reperfusion injury (IRI) or cecal ligation and puncture (CLP) to investigate the contribution of neutrophils to tissue injury and kidney function. By using VEC-Y731F mice, in which neutrophil recruitment is impaired, we analyzed the specific contribution of neutrophil recruitment to the pathogenesis of IRI- and CLP-induced AKI. We observed that the degree of renal injury evaluated by plasma creatinine, urinary biomarkers and histological analyses, following IRI-induction was dependent on neutrophil migration into the kidney, whereas the pathogenesis of CLP-induced AKI was independent of neutrophil recruitment. Furthermore, plasma transfer experiments suggest that the pathogenesis of CLP-induced AKI relies on circulating inflammatory mediators. These results extend our knowledge of the AKI pathogenesis and may help in the development of prophylactic and therapeutic treatments for AKI patients.

## Introduction

Acute kidney injury (AKI) is characterized by a sudden deterioration in renal function and is associated with an increased morbidity and mortality (1). AKI may be induced by different causes, including systemic inflammation, sepsis, shock, burn, trauma, and nephrotoxic agents (2). The etiologies of AKI can be divided into inflammatory (e.g. sepsis), ischemic (e.g. renal ischemia-reperfusion injury), and nephrotoxic causes (3). Previous reports postulated that sepsis-induced AKI is mainly based on renal ischemia. However, a growing body of evidence shows that renal blood flow is maintained or even increased under septic conditions (4) and despite occurring alterations in microvascular blood flow distribution, ischemia is not necessarily required for sepsis-induced kidney damage (5). Besides, inflammation, bioenergetic deficiencies and tubular cell adaptation are also involved in the pathogenesis of sepsis-induced AKI (6). The release of pathogen-associated molecular patterns (PAMPs) and damage-associated molecular patterns (DAMPs) into the circulation during sepsis initiates an inflammatory response, which further increases endothelial damage and tubular cell injury (7–9). Moreover, mitochondria-derived reactive oxygen species (ROS) production increases upon inflammatory stimulation, which is a sign of renal tubular damage caused by sepsis. ROS display an early event in septic conditions and are associated with renal areas showing intermittent or no blood flow (3, 10). Tubular metabolic down-regulation and cellular function reprioritization in response to pro-inflammatory processes and oxidative stress act as a negative feedback to limit further damage by maintaining energy balance and preventing further DNA damage *via* mitochondria (11–13). Therefore, systemic inflammation-induced mitochondrial dysfunction and ROS production in the kidney might dominate the pathogenesis of sepsis-induced AKI.

Renal ischemia/reperfusion injury (IRI) is a common clinical complication that may lead to the development of AKI (14). The central feature of renal IRI is tubular cell necrosis and apoptosis (15). After the ischemic insult, cell metabolism changes from aerobic to anaerobic, intracellular adenosine triphosphate (ATP) decreases, acidosis worsens, and membrane-bound Na^+^/K^+^ function decreases, leading to cellular edema and injury (16–18). During reperfusion, increased ROS production causes cell injury and apoptosis (19). Endogenous DAMPs released from injured and necrotic cells stimulate host cells to secrete pro-inflammatory and chemotactic cytokines, resulting in an influx of innate immune cells (3, 20). The innate immune response triggers and interacts with the adaptive immune system, therefore, both branches are involved in the pathology of IRI-induced AKI, including effector cells such as neutrophils, monocytes/macrophages, dendritic cells (DCs), natural killer (NK) cells, natural killer T (NKT) cells and lymphocytes (21). These processes lead to renal tubular cell impairment, glomerular filtration rate (GFR) decrease, and eventually AKI (22).

We hypothesized that neutrophil recruitment into the kidney tissue might play different roles in the pathogenesis of IRI-induced AKI compared to CLP-induced AKI. Therefore, we explored the effect of impaired neutrophil recruitment across the renal vascular endothelium on the pathogenesis of sepsis- and IRI-induced AKI by use of VEC-Y731F mice in this study. VEC-Y731F mice carry a point mutation at tyrosine 731 in VE-Cadherin that prevents the opening of endothelial cell junctions and specifically inhibits neutrophil recruitment from the vasculature into the perivascular tissue, while the induction of vascular permeability remains intact.

## Materials and Methods

### Animals and Reagents

We used 8–12 week old C57BL/6, and VEC-Y731F mice (23). The mice were kept in a barrier facility under specific pathogen-free (SPF) conditions. All animal experiments were approved by the IRB (LANUV NRW, approval number 81-02.04.2019.A182). Unless otherwise stated, all reagents were obtained from Sigma-Aldrich (Taufkirchen, Germany). Some mice received blocking antibodies against LFA-1 (clone TIB-217, i.v., 30 µg/mouse 15 min prior to surgery) and Mac1 (clone M1/70, i.v., 30 µg/mouse 15 min prior to surgery) or a neutrophil-depleting antibody (clone RB6-8C5, i.p., 10 µg/mouse 24 hours prior to surgery). In additional experiments plasma transfer samples were supplemented with blocking antibodies against CXCL1 and CXCL2 (4 µg/mouse, R&D Systems) (24).

### Renal Ischemia-Reperfusion Injury (IRI)

The IRI model has been described previously (25). Briefly, mice were anesthetized with intraperitoneal injections of ketamine (125 mg/kg) and xylazine (12.5 mg/kg) and placed on a heating pad to maintain body temperature. In animals undergoing IRI, kidneys were surgically exposed and both renal pedicles were clamped off for 35 min. After clamp removal, kidneys were checked for a change in color within 3 min to ensure reperfusion. In animals subjected to the sham operation, the surgical procedure was identical except that no clamps were applied. After surgery, animals were kept under a heating lamp to maintain body temperature and had free access to food and water.

### Cecal Ligation and Puncture (CLP)

CLP was performed as described before (26). Briefly, mice were anesthetized as mentioned above and a midline laparotomy incision was made after skin disinfection. The cecum was ligated 14 mm distal to the ileocecal valve to preserve continuity. Subsequently, the cecum was punctured twice with a 20-gauge needle and was returned to the peritoneal cavity. Incisions were closed and the mice were allowed to recover with free access to food and water. Animals in the sham group underwent the identical procedure without CLP. Animals did not receive resuscitation fluids or antibiotics. For blood plasma transfer experiments, CLP and sham surgery were induced in WT mice and whole blood was obtained by cardiac puncture in heparine-coated syringes after 24 hours. Plasma was isolated from whole blood by centrifugation for 10 min at 2000 g. Recipient mice were injected i.v. with 175 µl donor plasma per mouse.

### LPS-Induced AKI

AKI upon LPS induction was performed as described previously with minor modifications (27). Mice were intraperitoneally injected with PBS or LPS (10 µg/g body weight; O111:B4; Sigma). Following administration, animals were kept in home cages with free access to food and water and were sacrificed 24 hours after injection.

### Quantification of Renal Neutrophil Recruitment, Plasma Creatinine Levels and Biomarkers

After 24 h the mice were euthanized, and blood samples were obtained by cardiac puncture. After cutting off the vena cava, mice were perfused through the left ventricle and the flushing of kidneys was estimated by color change. Kidneys were harvested to determine the number of transmigrated neutrophils. Plasma creatinine levels were determined by using a creatinine assay kit (Diazyme, Poway, USA) according to the manufacturer’s protocol. Neutrophil recruitment into the kidneys was determined by flow cytometry as previously described (25). Briefly, after flushing and removal from the body, the kidneys were mechanically minced and enzymatically digested by incubation with collagenase, hyaluronidase and DNaseI. After 1 hour, the homogenized kidney tissue was passed over a cell strainer (mesh size 70 µm). Staining was performed with fluorescently-labeled antibodies against Gr-1 (clone 1A8), Ly6B.2 (clone 7/4) and CD45 (clone 30-F11) and samples were analyzed in a flow cytometer (BD FACSCantoII). Neutrophils were identified as CD45^+^Gr-1^+^Ly6B.2^+^ cells. Absolute neutrophil counts in the samples were determined by the use of fluorescent counting beads and analyzed with FlowJo software (version 7.6).

### Quantification of Urinary Biomarkers

The concentrations of NGAL, KIM-1, TIMP2 (all from R&D Systems) and IGFBP7 (Cusabio Technology LLC) were measured by using ELISA kits according to the manufacturers’ instructions.

### Luminex Analysis of Inflammatory Mediators

Plasma and urine samples were analyzed using the Cytokine & Chemokine 36-Plex Mouse ProcartaPlex Panel 1A (Thermo Fisher Scientific) run on a Luminex 200 analyzer (Luminex Corp.) according to the manufacturer’s instructions.

### Histological Examination

Kidneys were fixed in 10% formaldehyde, embedded in paraffin, and sectioned at 5 μm for hematoxylin & eosin (H&E), PAS and TUNEL staining. Histopathological examination was performed blinded to the conditions. Tubular injury was scored by estimating the percentage of tubules in the cortex that showed epithelial necrosis, loss of brush border, cast formation or tubular dilatation as follows: 0 = none; 1 = < 10%; 2 = 11–25%; 3 = 26–45%; and 4 = 46-75%; 5 = > 76% (28). For each slide, at least 10 fields were reviewed at a magnification of 20x. For histological analysis of cellular apoptosis we used the DeadEnd Colorimetric TUNEL System (Promega). Digital images of stained tissue sections (20x) were acquired by using a microscope with a digital camera (Lionheart FX Automated Microscope, BioTek). For each slice, 5 random fields were captured. TUNEL-positive cells were counted using ImageJ software v.1.3.7, and the percentage of apoptotic cells (TUNEL-positive cells) per field of view were calculated.

### Analysis of Neutrophil Phenotype

Differences in transmigrated neutrophils were assessed by flow cytometric analysis of common surface markers and sytox signals. Bone marrow, blood and enzymatically digested kidneys were stained with fluorescent antibodies (all from BioLegend) for neutrophils, which were defined as CD45^+^Ly6G^+^Ly6B.2^+^. Surface expression of CD11a (clone M7/14), CD11b (clone M1/70), CD54 (clone YN1/1.7.4), CD95 (clone SA367H8), CD62L (clone MEL-14), CD162 (clone 2PH1), CD182 (clone SA044G4), as well as Sytox^+^ cells (Thermo) were analyzed in a FACSCantoII flow cytometer (BD). Appropriate isotype antibodies were used for control stainings. Mean fluorescence intensities (MFIs) were identified with FlowJo software (version 7.6).

### Statistics

Statistical analysis was performed with SPSS (version 22.0) or GraphPad Prism (version 9) using Wilcoxon-test or t-test as appropriate. More than 2 groups were compared using one-way ANOVA followed by Bonferroni testing. Two-way repeated measures ANOVA was applied for more than two groups with two different categorical independent variables. Data distribution was assessed using Kolmogorov-Smirnov-test or Shapiro-Wilks test. All data are represented as mean ± SEM. A p-value < 0.05 was considered statistically significant. For *in vivo* experiments, the provided *n* is the number of animals used per experiment.

## Results

### The Pathogenesis of IRI-Induced AKI Critically Involves Neutrophil Recruitment

**Figure 1:**
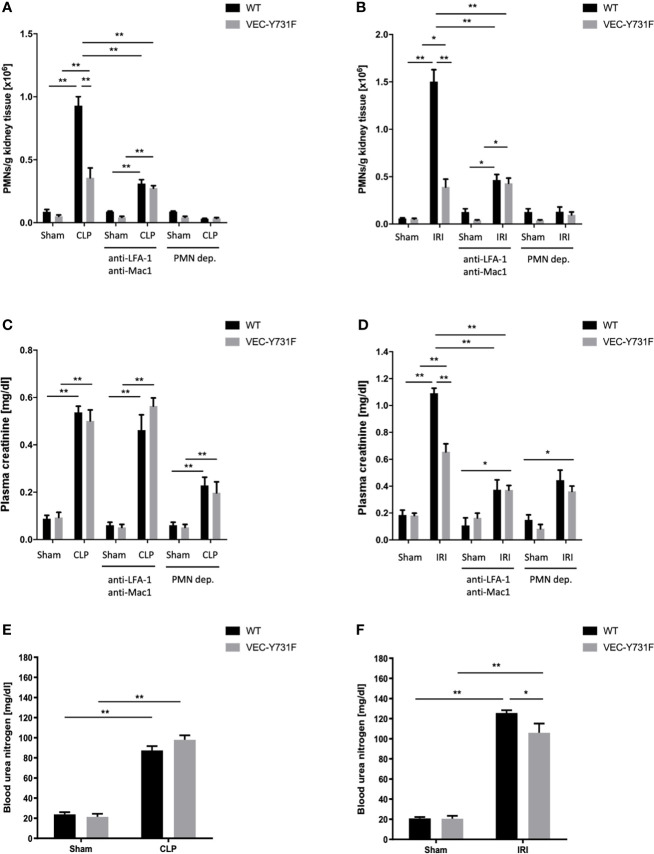
The pathogenesis of IRI-induced AKI critically involves neutrophil recruitment. AKI in WT and VEC-Y731F mice was induced either by CLP or IRI. Some mice received blocking antibodies against LFA-1 (clone TIB-217, 30 µg/mouse 15 min prior to surgery) and Mac1 (clone M1/70, 30 µg/mouse 15 min prior to surgery) or a neutrophil-depleting antibody (clone RB6-8C5, 10 µg/mouse 24 hours prior to surgery). 24 hours after AKI-induction by CLP **(A)** or IRI **(B)** surgery neutrophil recruitment into the kidney was analyzed by flow cytometry. Plasma creatinine after AKI-induction by CLP **(C)** or IRI **(D)** surgery and blood urea nitrogen after AKI-induction by CLP **(E)** or IRI **(F)** surgery were analyzed by photometry 24 hours after the procedure (n=8-12). Data are mean ± SEM. **p < 0.01, *p < 0.05.

Both the IRI procedure and CLP surgery caused neutrophil recruitment into the kidneys after 24 hours in WT mice (**Figures 1A, B** and **Supplemental Figure 1**). In VEC-Y731F mice, neutrophil recruitment into the renal tissue was significantly decreased following both IRI and CLP (**Figures 1A, B** and **Supplemental Figure 1**). Interestingly, impaired neutrophil recruitment in VEC-Y731F mice resulted in decreased plasma creatinine concentrations as a surrogate parameter of renal function after induction of AKI by IRI compared to WT mice, whereas the impaired neutrophil recruitment in VEC-Y731F following CLP surgery did not affect plasma creatinine concentrations (**Figures 1C, D**). The neutrophil integrins LFA-1 and Mac1 are known to be required for neutrophil recruitment into the kidney during CLP-induced AKI (29). The administration of blocking antibodies against LFA-1 and Mac1 prior to CLP or IRI surgery leads to decreased neutrophil recruitment into the kidney (**Figures 1A, B** and **Supplemental Figure 1**). Blocking both integrins significantly reduced plasma creatinine levels after IRI, but not after CLP (24 hours after the procedures; **Figure 1C, D**). Neutrophil depletion prior to CLP or IRI surgery significantly reduced neutrophil recruitment into the kidney and plasma creatinine levels in both animal models (**Figures 1A–D** and **Supplemental Figure 1**). Decreased blood urea nitrogen (BUN) levels were only observed in VEC-Y731F mice after induction of AKI by IRI, but not after induction of AKI by CLP (**Figures 1E, F**). To further substantiate our findings, we investigated the influence of transmigrated neutrophils on the induction of AKI in an additional sepsis model. Intraperitoneally injection of 10 µg/g body weight LPS resulted in elevated PMN recruitment into the kidneys and heightened plasma creatinine levels in WT mice (**Supplemental Figures 2A, B**). Comparable with the observations in CLP mice, PMN recruitment in VEC-Y731F mice was significantly decreased compared to WT mice, while the level of kidney injury, as specified by plasma creatinine, remained clearly enhanced (**Supplemental Figures 2A, B**). Peripheral blood neutrophil counts did not differ between the different mouse genotypes and interventions (**Supplemental Figure 3**). These data indicate that neutrophils are important for AKI development following sepsis and IRI. However, the pathogenesis of AKI involves neutrophil migration into the kidneys following IRI, whereas the pathogenesis of sepsis-induced AKI does not critically involve neutrophil recruitment into the kidneys.

### Impaired Neutrophil Recruitment Decreases Urinary Concentrations of Renal Injury Biomarkers Only in IRI- but Not CLP-Induced AKI

To further characterize the degree of AKI in mice, we measured the urinary concentration of common AKI biomarkers in WT and VEC-Y731F mice 24 hours after IRI- or CLP-induction (**Supplemental Figure 4**). Strikingly, the urinary concentrations of NGAL, KIM-1, TIMP2 and IGFBP7 were significantly decreased in VEC-Y731F mice upon IRI-induced AKI compared to WT mice (**Supplemental Figures 4B, D, F, H**). However, the biomarker levels were similar in WT mice and VEC-Y731F mice upon CLP-induced AKI (**Supplemental Figures 4A, C, E, G**). These data offer further compelling evidence that renal injury as judged by the measurement of urinary biomarkers is primarily dependent on neutrophil recruitment in IRI-induced AKI and not following CLP-induced AKI.

### Impaired Neutrophil Recruitment Decreases Renal Tissue Injury and Cell Apoptosis Only Following IRI- but Not CLP-Induced AKI

**Figure 2:**
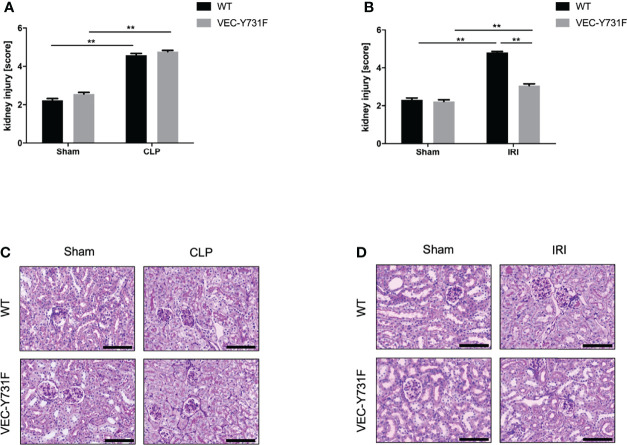
Impaired neutrophil recruitment decreases renal tissue injury and cell apoptosis only in IRI- but not CLP-induced AKI. AKI in WT and VEC-Y731F mice was induced either by CLP or IRI. 24 hours after AKI-induction renal cortical tissue sections were excised, fixed in formaldehyde, embedded in paraffin and processed for H&E staining. The histological analysis was performed from at least 25 high-power fields per kidney from WT and VEC-Y731F mice after CLP **(A)** or IRI **(B)** surgery and the kidney injury score was calculated. Exemplary histological images of H&E staining from WT and VEC-Y731F mice after CLP **(C)** or IRI **(D)** surgery (n = 3). Data are mean ± SEM. **p < 0.01. Scale bar: 100 µm.

We performed histological analysis of cortical renal tissue injury and subsequent histological scoring of kidney sections obtained from WT and VEC-Y731F mice 24 hours after AKI-induction by IRI or CLP procedure. While AKI-induction upon both IRI and CLP surgery caused substantial histologically apparent renal tissue injury in WT mice, VEC-Y731F mice showed a substantial alleviation of histological renal tissue damage after IRI surgery, but not after CLP surgery (**Figures 2A–D**). Furthermore, we analyzed the abundance of apoptotic cells in renal tissue sections by TUNEL staining. These experiments also revealed that impaired neutrophil recruitment in VEC-Y731F mice was associated with a significantly decreased rate of TUNEL-positive (apoptotic) cells in the renal tissue only in IRI-induced, but not after CLP-induced AKI (**Supplemental Figures 5A–D**). Thus, these data support our findings that the degree of renal tissue injury and the rate of cellular apoptosis in renal tissue cells is predominantly neutrophil-dependent in the pathogenesis of IRI-induced AKI, but not during CLP-induced AKI.

### ROS Levels in Kidney Tissue Are Decreased in Mice With Impaired Neutrophil Recruitment Only Following IRI-Induced AKI, but Not CLP-Induced AKI

**Figure 3:**
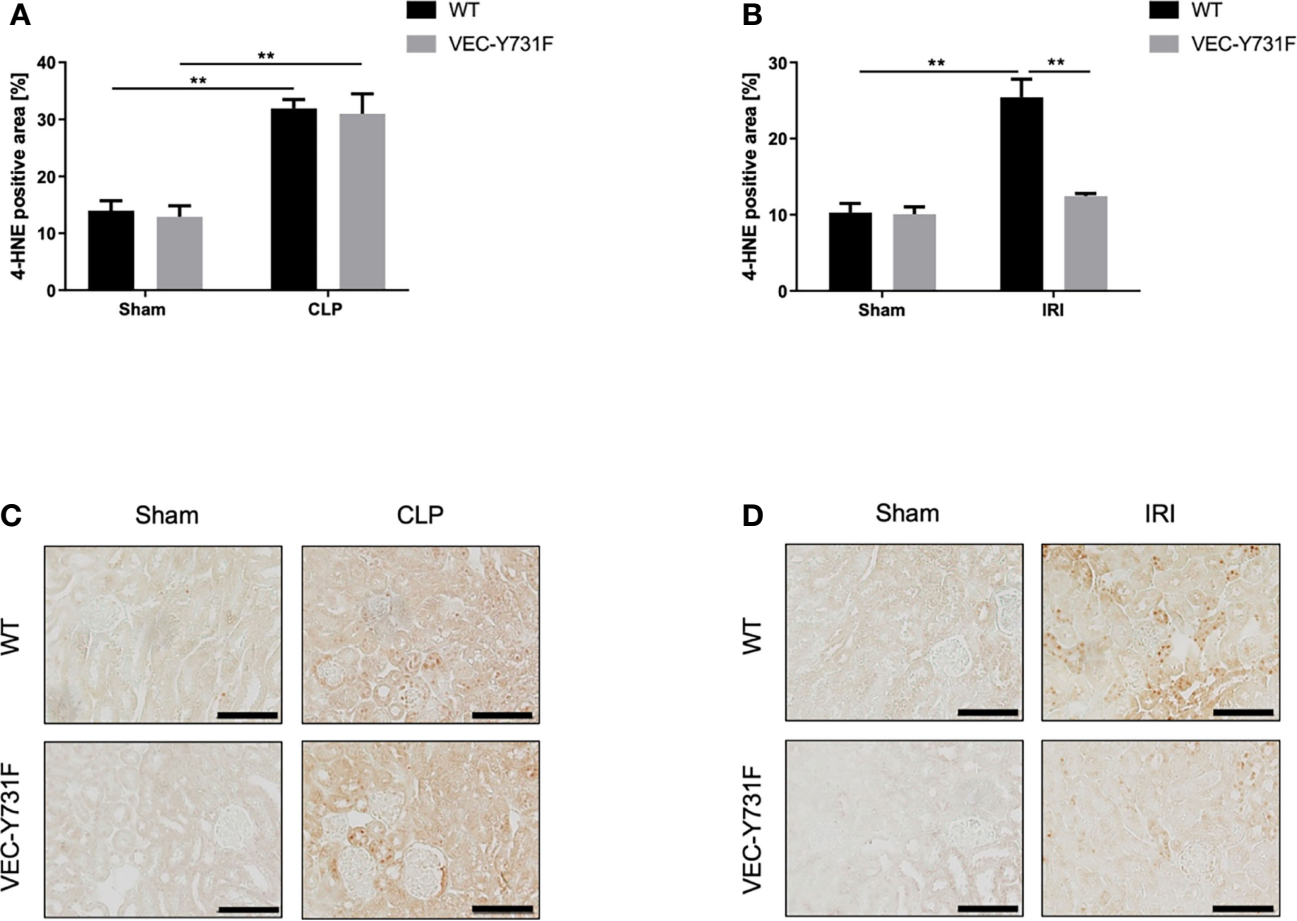
ROS levels in kidney tissue are decreased in mice with impaired neutrophil recruitment only following IRI-induced AKI, but not CLP-induced AKI. AKI in WT and VEC-Y731F mice was induced either by CLP or IRI. 24 hours after AKI-induction renal cortical tissue sections were excised, fixed in formaldehyde, embedded in paraffin and processed for immunohistochemistry staining against 4-HNE to quantify tissue ROS levels. The histological analysis was performed from at least 25 high-power fields per kidney from WT and VEC-Y731F mice after CLP **(A)** or IRI **(B)** surgery and the 4-HNE-positive tissue area was quantified by computational image analysis. Exemplary histological images of 4-HNE staining from WT and VEC-Y731F mice after CLP **(C)** or IRI **(D)** surgery (n = 3). Data are mean ± SEM. **p < 0.01. Scale bar: 100 µm.

Oxidative stress is an important driving factor in the pathophysiology of AKI, thus, we analyzed ROS levels in the renal tissue after CLP- and IRI-surgery by immunohistochemical anti-4HNE staining. The induction of AKI both by CLP and IRI surgery caused a detectable increase in ROS levels in the renal tissue in WT mice compared to sham-operated mice (**Figures 3A–D**). However, ROS levels in the renal tissue were significantly lower in VEC-Y731F mice after IRI surgery compared to WT mice (**Figures 3B, D**), whereas ROS levels in the tissue of VEC-Y731F mice after CLP were similar to the levels of WT mice after CLP (**Figures 3A, C**). Interestingly, CLP triggered higher ROS levels in the plasma and urine of WT mice compared to IRI surgery (**Supplemental Figures 6A, B**). These results suggest that impaired neutrophil recruitment alleviates ROS levels in renal tissue following IRI- but not CLP-induced AKI.

### The Pathogenesis of CLP-Induced AKI Relies on Pro-Inflammatory Mediators

**Figure 4:**
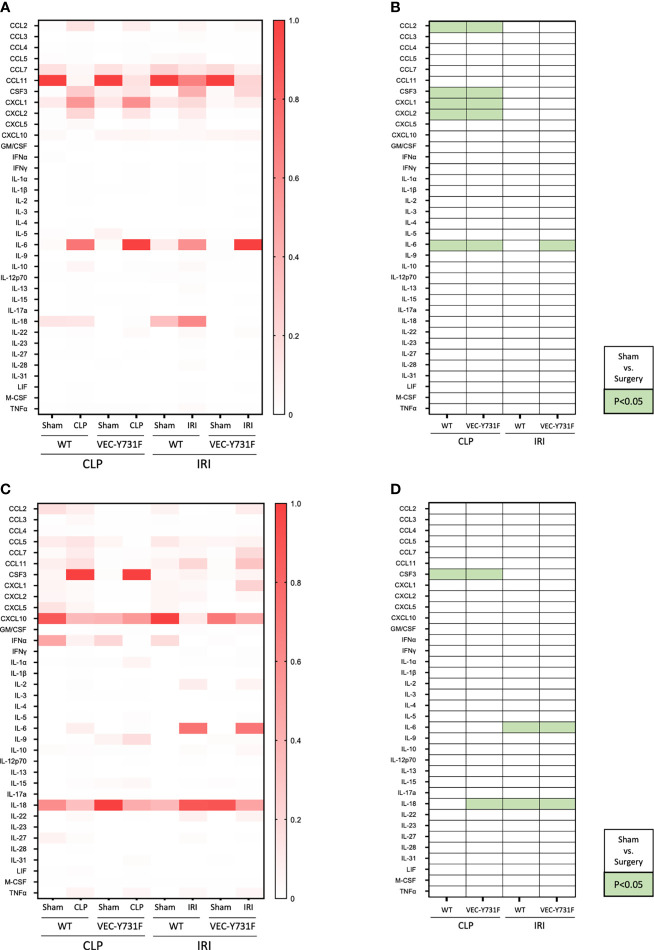
CLP- and IRI-induced AKI is associated with different patterns of inflammatory mediators in plasma and urine. AKI in WT and VEC-Y731F mice was induced either by CLP or IRI. 24 hours after AKI-induction the concentrations of inflammatory mediators in the plasma **(A, B)** and the urine **(C, D)** were analyzed by multiplex analysis including a panel of 36 different analytes (n=5-12). Visualization was performed by normalizing each subcolumn separately and defining the largest value in each subcolumn as 100% using Prism built-in analysis. Heat map data are presented as fractions for plasma **(A)** and urine **(C)** samples. Significant differences (p < 0.05) between sham and surgery groups are displayed in green for plasma **(B)** and urine **(D)** samples (n = 5-12).

So far, our experimental data implicate that the pathogenesis of IRI-induced AKI critically relies on the recruitment of neutrophils into the renal tissue, whereas the pathogenesis of CLP-induced AKI does not. We performed a multiplex analysis of soluble pro- and anti-inflammatory mediators in the plasma and urine of WT and VEC-Y731F mice 24 hours after initiation of CLP- and IRI-induced AKI. The induction of CLP caused a pronounced increase of inflammatory mediators in the plasma, including CCL2, CSF3, CXCL1, CXCL2 and IL-6 compared to sham controls in both WT and VEC-Y731F mice. This effect was not apparent after IRI surgery (**Figures 4A, B**). CSF3 was additionally found to be evaluated not only in plasma but also in urine samples of WT and VEC-Y731F mice after CLP. IL-6 and IL-18, however, were increased upon IRI surgery in urine samples of both mice strains (**Figures 4C, D**).

**Figure 5:**
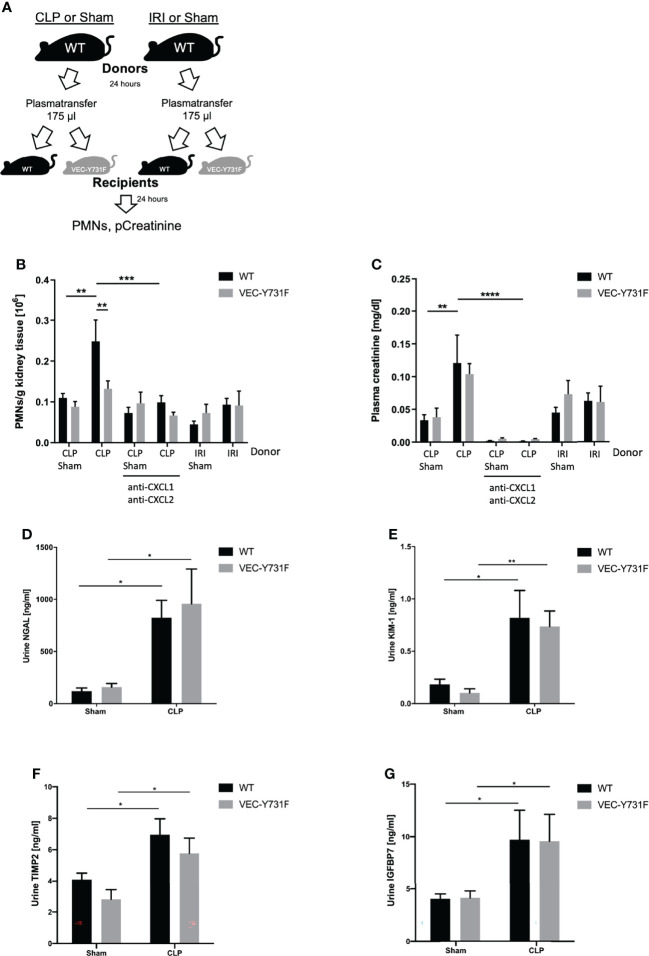
The pathogenesis of CLP-induced AKI relies on proinflammatory mediators. Sham, CLP, or IRI-surgery was performed in WT mice and blood samples for plasma isolation were obtained after 24 hours. 175 µl plasma from sham-, IRI- or CLP-operated WT mice was injected intravenously into WT and VEC-Y731F mice. **(A)** Illustrative scheme of plasma transfer experiments. 24 hours after plasma injection the neutrophil recruitment into the kidneys was analyzed by flow cytometry **(B)** and plasma creatinine concentrations were analyzed by photometry **(C)**. CLP transfer experiments were performed with supplemented anti-CXCL1 and anti-CXCL2 (4 µg/mouse, R&D Systems) The concentrations of the biomarkers NGAL **(D)**, KIM-1 **(E)**, TIMP2 **(F)** and IGFBP7 **(G)** were measured by ELISA (n = 4-9). Data are mean ± SEM. ****p < 0.0001, ***p < 0.001, **p < 0.01, *p < 0.05.

To further investigate this observation and to analyze if the development of CLP-induced AKI relies on soluble, pro-inflammatory mediators in the blood we performed plasma transfer experiments. For this purpose, blood was harvested from WT mice 24 hours after sham, CLP or IRI surgery. The obtained plasma was injected intravenously into WT and VEC-Y731F mice (**Figure 5A**). The transfer of plasma from CLP mice induced a noticeable recruitment of neutrophils in WT, but not in VEC-Y731F recipient mice (**Figure 5B**). Interestingly, the transfer of plasma from IRI mice in either WT or VEC-Y731F mice did not cause neutrophil recruitment into the kidney or increased plasma creatinine levels, indicating that the pathogenesis of IRI-induced AKI does not rely on plasma-derived soluble mediators (**Figures 5B, C**). However, regardless of impaired neutrophil recruitment in VEC-Y731F mice, the degree of resulting kidney injury in WT and VEC-Y731F recipient mice infused with plasma from CLP mice was similar according to plasma creatinine (**Figure 5C**) and the urinary AKI biomarkers NGAL, KIM-1, TIMP2 and IGFBP7 (**Figures 5D–G**). Likewise, the renal tubular cell apoptosis as quantified by TUNEL, as well as histological injury analyzed by H&E and PAS stainings of renal tissue sections from WT and VEC-Y731F recipient mice infused with plasma from CLP mice was similar after 24 hours (**Supplemental Figures 7A–E**). Since the analysis of pro-inflammatory mediators suggested a potential role for CXCL1 and CXCL2 in the manifestation of CLP-induced AKI, we supplemented plasma from CLP mice with appropriate blocking antibodies right before its transfer into recipient mice. The supplementation of CLP plasma samples with blocking antibodies against CXCL1 and CXCL2 clearly reversed previous effects. Neither PMN recruitment nor altered plasma creatinine levels could be detected in recipient mice, thereby supporting the importance of these chemokines in CLP-induced AKI (**Figures 5B, C**).These data support our hypothesis that the pathogenesis of kidney injury during systemic inflammation following CLP surgery relies on the presence of soluble, pro-inflammatory mediators and, apparently, is independent of neutrophil migration into the kidneys, whereas the pathogenesis of IRI-induced AKI critically involves neutrophil recruitment into the renal tissue.

### The Mode of AKI Shapes the Phenotype of Recruited Neutrophils

**Figure 6:**
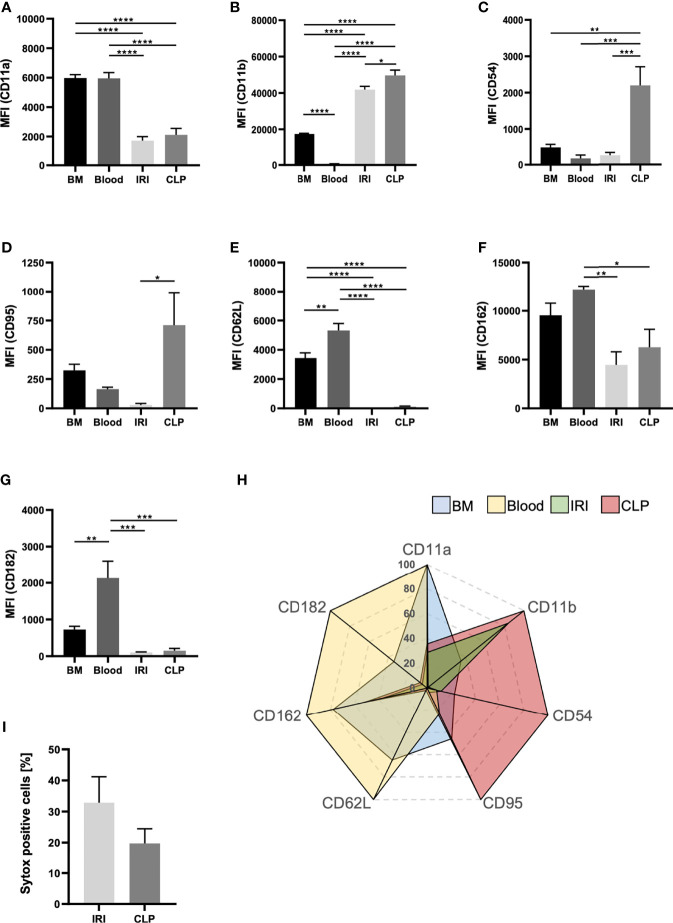
The mode of AKI shapes the phenotype of recruited neutrophils. AKI was induced in WT mice *via* IRI or CLP. Transmigrated neutrophils, defined as CD45^+^Ly-6G^+^Ly-6B.2^+^, from kidney tissue were analyzed for classical surface markers. Bone marrow and blood neutrophils were analyzed from healthy, adult WT mice. Mean fluorescence intensities (MFIs) of CD11a **(A)**, CD11b **(B)**, CD54 **(C)**, CD95 **(D)**, CD62L **(E)**, CD162 **(F)** and CD182 **(G)** on bone marrow-, blood- IRI- and CLP-derived neutrophils were determined by flow cytometry (n = 5). Sytox positive cells are shown in percentages of total cells **(H)** (n = 5). Clustering of neutrophil phenotype is displayed in a radar plot **(I)**. Data are mean ± SEM. ****p < 0.0001, ***p < 0.001, **p < 0.01, *p < 0.05.

Since neutrophils transmigrate into the kidneys upon IRI and CLP, but primarily promote AKI upon IRI, we induced AKI *via* IRI and CLP in WT mice and analyzed transmigrated neutrophils from the kidneys *via* flow cytometry to detect potential differences in neutrophil characteristics (**Supplemental Figure 8**). We investigated classical neutrophil surface markers and compared not only neutrophils from IRI and CLP mice, but also neutrophils derived from healthy bone marrow and peripheral blood. Surface expression of CD11a, CD62L and CD182 on neutrophils significantly decreased after induction of IRI or CLP compared to bone marrow- and blood-derived neutrophils, but the AKI-induction method did not provoke extensive differences (**Figures 6A, E, G**). However, CLP surgery resulted in elevated surface expression of CD11b, CD54 and CD95 in neutrophils, while IRI provoked only mild or no increases (**Figures 6B, C, D**). Surface availability of neutrophil CD162 was not affected by IRI or CLP compared to bone-marrow derived neutrophils but was significantly decreased in contrast to blood-derived neutrophils (**Figure 6F**). Finally, we examined the potential of IRI and CLP neutrophils to release neutrophil extracellular traps (NETs) by analyzing sytox signals *via* flow cytometry. The results point to a trend, that neutrophils transmigrated to kidneys upon IRI are more prone to release NETs than CLP-driven neutrophils (**Figure 6H**). Taken together, induction of IRI and CLP promotes distinct neutrophil phenotypes, which clearly differ among each other and differ from untreated bone marrow- and blood-derived neutrophils (**Figure 6I**).

## Discussion

This study investigated the role of neutrophil recruitment in sepsis- and IRI-induced AKI by using murine models of CLP and IRI. Our data provide hints that while the presence of circulating neutrophils is necessary to induce AKI upon sepsis, neutrophil recruitment into the kidneys is not critical for CLP- or LPS-induced AKI. Thus, the data from our study suggest that neutrophil recruitment may play different roles during the pathogenesis of AKI induced by ischemia-reperfusion or systemic inflammation.

During sepsis- and IRI-induced AKI, the release of DAMPs and PAMPs activates monocytes/macrophages and dendritic cells to secrete pro-inflammatory cytokines/chemokines and subsequently initiates the recruitment of neutrophils into the renal tissue (7, 20, 30). While during IRI, DAMPs may be released after endogenous cell injury, PAMPs are released from exogenous bacteria during sepsis into the circulation. After intravascular activation, neutrophils may exert antimicrobial effects, but also contribute to excessive collateral kidney tissue damage (31). Thus, activated neutrophils may promote the inflammatory response in AKI (32, 33). This is also in accordance with our observation that neutrophils are crucially involved in the pathogenesis of both CLP- and IRI-induced AKI as systemic neutrophil depletion protected, at least partially, from AKI development in both models of AKI. The inhibition of neutrophil recruitment into the renal tissue either by use of blocking antibodies or VEC-Y731F mice only protected the mice from IRI-induced AKI, but not from CLP-induced AKI. Thus, in contrast to IRI-induced AKI, the pathogenesis of CLP-induced AKI is mainly driven by the intravascular presence of neutrophils, but not by their recruitment into the tissue. This may be explained by the participation of soluble pro-inflammatory mediators, neutrophil-derived ROS and activated endothelial cells. ROS-mediated cellular toxicity results from lipid peroxidation, direct DNA damage, membrane damage, and the production of other free radicals (34, 35). During ischemia minor amounts of ROS are generated from mitochondria in diverse renal cell types due to oxygen deprivation and ATP shortage (36). This is also reflected by our results where IRI-induced AKI was associated with only minor elevations in plasma and urine ROS levels. This, however, is different during systemic inflammatory processes where excessive ROS release by different inflammatory cell subsets (e.g., neutrophils, macrophages, NK cells) characterizes the inflammatory response and may result in peripheral organ damage driving kidney injury and dysfunction (19, 34, 37).

In addition to the release of ROS, an uncontrolled systemic inflammation leads to increased circulating levels of inflammatory cytokines (38). Of note, cellular ROS production and inflammatory cytokine release may be enhanced by each other during the disease progression (39). This is also reflected by the results of our systematic screen of inflammatory mediators in the plasma and urine of mice after initiation of CLP- or IRI-induced AKI. Here, CLP-induced AKI was accompanied by sharply elevated levels of classical pro-inflammatory chemokines and cytokines like CXCL1, CXCL2 and CSFR mainly in the plasma of mice. Mechanistically, the action of inflammatory mediators during systemic inflammation may cause blood flow redistribution between the renal medulla and renal cortex and subsequently medullary ischemia, which may contribute to the pathophysiology of sepsis-induced AKI (40). Even more importantly, the tubular metabolism with decreased ATP supply and mitochondrial dysfunction due to the kidney regional circulation alteration has been identified as a new critical mechanism in sepsis-induced AKI (41). Here, inflammatory cytokines promote mitochondrial oxidant production, which could further impair mitochondrial biogenesis, including reduced ATP production and bioenergetic failure, leading to renal dysfunction and damage (42, 43). The impact of CXCL1 and CXCL2 on AKI development was highlighted when appropriate blocking antibodies attenuated the effect of plasma transferred from septic mice on the manifestation of AKI in recipients. CXCL1 and CXCL2 are critical chemokines for the recruitment and activation of neutrophils. Neutrophils were found to follow a CXCL1 and/or CXCL2 gradient to sites of infection and stimulation further results in the release of proteases and ROS (44, 45). Thereby, uncontrolled levels of CXCL1 and CXCL2 might cause augmented tissue injury, by overactivating neutrophils. However, blocking of CXCL1 and CXCL2 might also be accompanied by diminished host response (46).

During IRI-induced AKI we observed a different situation. Interestingly, the pro-inflammatory cytokines IL-6 and IL-18 were elevated only in the urine after IRI surgery. Using VEC-Y731F mice, we demonstrated that inhibition of neutrophil extravasation abolished the neutrophil-driven “inflammatory burn” triggered by ROS and ameliorated IRI-induced AKI (23). This was also supported by the results from our plasma transfer experiments in which the injection of plasma from donor mice after initiation of IRI-induced AKI did not induce significant kidney injury in WT recipient mice. During IRI-induced AKI, neutrophils and endothelial cells are activated by ischemia and reperfusion, generating pro-inflammatory cytokines such as IL-1β, TNF-α, IL-6 and IL-18, inducing neutrophils adhesion, degranulation, and activation of the NADPH oxidase (47) Interestingly, IL-6 and IL-18 were significantly increased in our urine samples after inducing renal IRI. IL-18 is a well-known player in the manifestation of IRI-induced AKI. Wu et al. noticed that IL-18 affects kidney function and tubular damage and that bone marrow-derived cells are the main players in IL-18-mediated effects (48). Finally, it was shown that IL-18 inhibition reduces kidney injury, which is in line with our findings (49). There is evidence in the literature that neutrophils are involved in the pathogenesis of IRI-induced AKI from early to late stages (3). Thus, our results suggest that soluble pro-inflammatory mediators indirectly drive remote organ injury during systemic inflammation, in this case CLP, whereas the pathogenesis of IRI-induced AKI relies more on direct, cellular-mediated effects in the affected renal tissue itself.

Not only pro-inflammatory mediators, but also the possibility of distinct neutrophil phenotypes aroused our interest in the context of AKI-induction. Neutrophils were found to specialize in relation to immunoregulatory functions (50). The N1 and N2 phenotypes for example are of special interest in cancer research (51–53). N1 neutrophils are considered anti-tumorigenic, while N2 are rather pro-tumorigenic and differ in the extent of exerting effector functions (53). In a comprehensive screening of common neutrophil surface markers, we identified neutrophils derived from kidneys of CLP mice as CD11b^high^, CD54^high^ and CD95^high^, thus, pointing towards an N1 phenotype (50, 53). On the contrary, neutrophils recruited to kidney after IRI surgery show lower levels of CD11b, CD54 and CD95. The expression of CD54 in neutrophils is increased upon stimulation with LPS and correlates with enhanced phagocytosis and ROS generation (54), while CD95 is known for its contributions to neutrophil slow rolling and adhesion (55). Furthermore, compared to neutrophils derived from IRI mice, CLP-derived neutrophils showed a decreased number of sytox positive cells. The nucleic-acid dye sytox is impermeable for intact living cells and widely used as an indicator for NET formation (56). NETs are web-like structures equipped with several anti-microbial substances and released by activated neutrophils to capture and combat pathogens. However, excessive NET release results in exceeding damage of host tissues (57). These results emphasize that sepsis and IRI promote different features in neutrophils. The underlying mechanisms, however, remain to be elucidated.

Previous studies show that reduced neutrophil counts or inflammatory cytokines are partly related to the attenuation of kidney injury (58–61). However, most of these studies relied on methods that cause decreases in systemic neutrophil counts or reduced inflammatory cytokines, but not the direct inhibition of neutrophil recruitment into the renal tissue. In our study, renal injury during systemic inflammation was not significantly alleviated after specifically targeting neutrophil recruitment into the kidney leaving the remaining neutrophils inside the vessel unaltered and fully functional. These results suggest that the uncontrolled systemic inflammatory response, but not local neutrophil recruitment into the kidney, dominates the pathogenesis of kidney injury in sepsis-induced AKI. In contrast, however, local neutrophil recruitment into the kidney tissues appears to play an amplifying role in the process of IRI-induced AKI, possibly involving local tissue reprogramming and destruction.

Although our study proposes that the progressive factors are infiltrated neutrophils and pro-inflammatory mediators in IRI-induced AKI and CLP-induced AKI, respectively, AKI is a complex process involving multiple factors, and some limitation should be noted. Firstly, in IRI-induced AKI, the specific mechanism inducing kidney damage by infiltrating neutrophils was not explored, and the impact of other immune cells, such as macrophages, and the types and proportions of immune cells involved in AKI remain unclear. Secondly, in CLP-induced AKI, the specific mechanisms of single circulating proinflammatory mediators in renal injury remains to be addressed.

## Data Availability Statement

The original contributions presented in the study are included in the article/**Supplementary Material**. Further inquiries can be directed to the corresponding author.

## Ethics Statement

The animal study was reviewed and approved by LANUV NRW.

## Author Contributions

ZL, NL, KT, SM and ML: performed the experiments and analyzed the data; JR: performed experiments, wrote the manuscript and designed the experiments: ZL, NL and HG: also contributed to writing the manuscript; JK: conceived of the mechanism and contributed to writing the manuscript; DV and JFP: provided materials and contributed to writing the manuscript; AZ conceived of the study and contributed to writing the manuscript. All authors contributed to the article and approved the submitted version.

## Funding

This work was supported by the Center for Interdisciplinary Clinical Research (IZKF Münster, Ross2/010/18 to JR) and the Deutsche Forschungsgemeinschaft (RO4537/4-1 to JR and ZA428/17-1, SFB1009A05 and TRR332C01 to AZ).

## Conflict of Interest

The authors declare that the research was conducted in the absence of any commercial or financial relationships that could be construed as a potential conflict of interest.

## Publisher’s Note

All claims expressed in this article are solely those of the authors and do not necessarily represent those of their affiliated organizations, or those of the publisher, the editors and the reviewers. Any product that may be evaluated in this article, or claim that may be made by its manufacturer, is not guaranteed or endorsed by the publisher.

## Supplementary Material

Supplementary Figure 1 Flow cytometry gating strategy for the determination of neutrophil counts after CLP and IRI. AKI was induced in WT and VEC-Y731F mice by CLP or IRI. Sham animals served as appropriate controls. Kidneys were enzymatically digested, and neutrophils were stained with CD45-PerCP/Cy5.5 (clone 30-F11), Gr-1-AF633 (clone 1A8) and Ly6B.2-FITC (clone 7/4). Cells were chosen by FSC-A/SSC-A size discrimination. CD45^+^-leukocytes were finally examined for a Gr-1^+^Ly6B.2^+^ population. The double positive population was considered as neutrophils and total counts were determined for further calculations.

Supplementary Figure 2 LPS-induced sepsis results in AKI independent of neutrophil recruitment. WT and VEC-Y731F mice were injected with 10 µg LPS/ g body weight. Neutrophil recruitment into the kidneys **(A)** and plasma creatinine **(B)** were analyzed 24 h after injection. WT mice injected with PBS served as sham controls (n=5). Data are mean ± SEM. ***p < 0.001, **p < 0.01.

Supplementary Figure 3 Peripheral blood granulocyte counts. Whole blood samples were obtained from WT and VEC-Y731F mice before CLP- **(A)** or IRI-surgery **(B)**. The number of granulocytes in peripheral blood was analyzed by kimura staining using a modified Neubauer chamber (n=6-10). Data are mean ± SEM.

Supplementary Figure 4 Impaired neutrophil recruitment decreases urinary concentration of renal injury biomarkers only in IRI- but not CLP-induced AKI. AKI in WT and VEC-Y731F mice was induced either by CLP or IRI. 24 hours after AKI-induction by CLP- or IRI-surgery the concentrations of the biomarkers NGAL **(A, B)**, KIM-1 **(C, D)**, TIMP2 **(E, F)** and IGFBP7 **(G, H)** were measured by ELISA (n=3-8). Data are mean ± SEM. **p < 0.01, *p < 0.05.

Supplementary Figure 5 Impaired neutrophil recruitment decreases renal tissue apoptosis only in IRI- but not CLP-induced AKI. AKI in WT and VEC-Y731F mice was induced either by CLP or IRI. 24 hours after AKI-induction by CLP- or IRI-surgery renal cortical tissue sections were excised, fixed in formaldehyde, embedded, and processed for TUNEL stainings. The histological analysis was performed from at least 25 high-power fields per kidney from WT and VEC-Y731F mice after CLP- **(A)** or IRI-surgery **(B)** and the percentage of TUNEL-positive cells was counted. Exemplary histological images of TUNEL stainings from WT and VEC-Y731F mice after CLP- **(C)** or IRI-surgery **(D)** (n=3). Data are mean ± SEM. **p < 0.01. Scale bar: 100 µm.

Supplementary Figure 6 ROS levels in plasma and urine after AKI-induction by CLP and IRI. Plasma and urine samples were obtained from WT mice 24 hours after induction of CLP- or IRI-induced AKI. ROS levels in plasma **(A)** and urine **(B)** were analyzed by a photometric assay (n=4-5). Data are mean ± SEM. **p < 0.01

Supplementary Figure 7 The pathogenesis of CLP-induced AKI relies on pro-inflammatory mediators. Sham or CLP-surgery was performed in WT mice. Plasma samples were obtained after 24 hours and 175 µl plasma from sham- or CLP-operated WT mice was injected intravenously into WT and VEC-Y731F mice. After 24 hours renal cortical tissue sections were excised, fixed in formaldehyde, embedded in paraffin, and processed for TUNEL **(A, B)**, H&E **(C, D)** and PAS **(E)** stainings. The histological analysis was performed from at least 25 high-power fields per kidney from WT and VEC-Y731F mice after receiving donor plasma from sham- or CLP-operated WT mice. Exemplary images of TUNEL, H&E and PAS stainings **(B, D, E)** (n=4-5). Data are mean ± SEM. **p < 0.01, *p < 0.05. Scale bar: 100 µm.

Supplementary Figure 8 Flow cytometry gating strategy for the determination of neutrophil characteristics. Neutrophils were derived from bone marrow and blood of healthy mice. AKI was induced in WT and VEC-Y731F mice by CLP or IRI and kidneys were enzymatically digested. Samples were stained with fluorescent antibodies and analyzed in a FACSCantoII flow cytometer (BD). Cells were chosen by FSC-A/SSC-A and FSC-H/FSC-A size and singlets discrimination, respectively. CD45^+^Ly-6G^+^Ly6B.2^+^ populations were considered as neutrophils and analyzed for Pe signals. Representative gating strategies for bone-marrow-derived **(A)**, peripheral blood-derived **(B)** and kidney-derived **(C)** neutrophils. Sytox^+^ cells were chosen from a Ly-6G^+^ population. Light orange: isotype control; deep orange: antibody staining.
