# Supplementary figures and images for: The Pathogenesis of Ischemia-Reperfusion Induced Acute Kidney Injury Depends on Renal Neutrophil Recruitment Whereas Sepsis-Induced AKI Does Not
Source: Front Immunol. 2022 Apr 21;13:843782. doi: 10.3389/fimmu.2022.843782 (PMC9069608; doi:10.3389/fimmu.2022.843782)

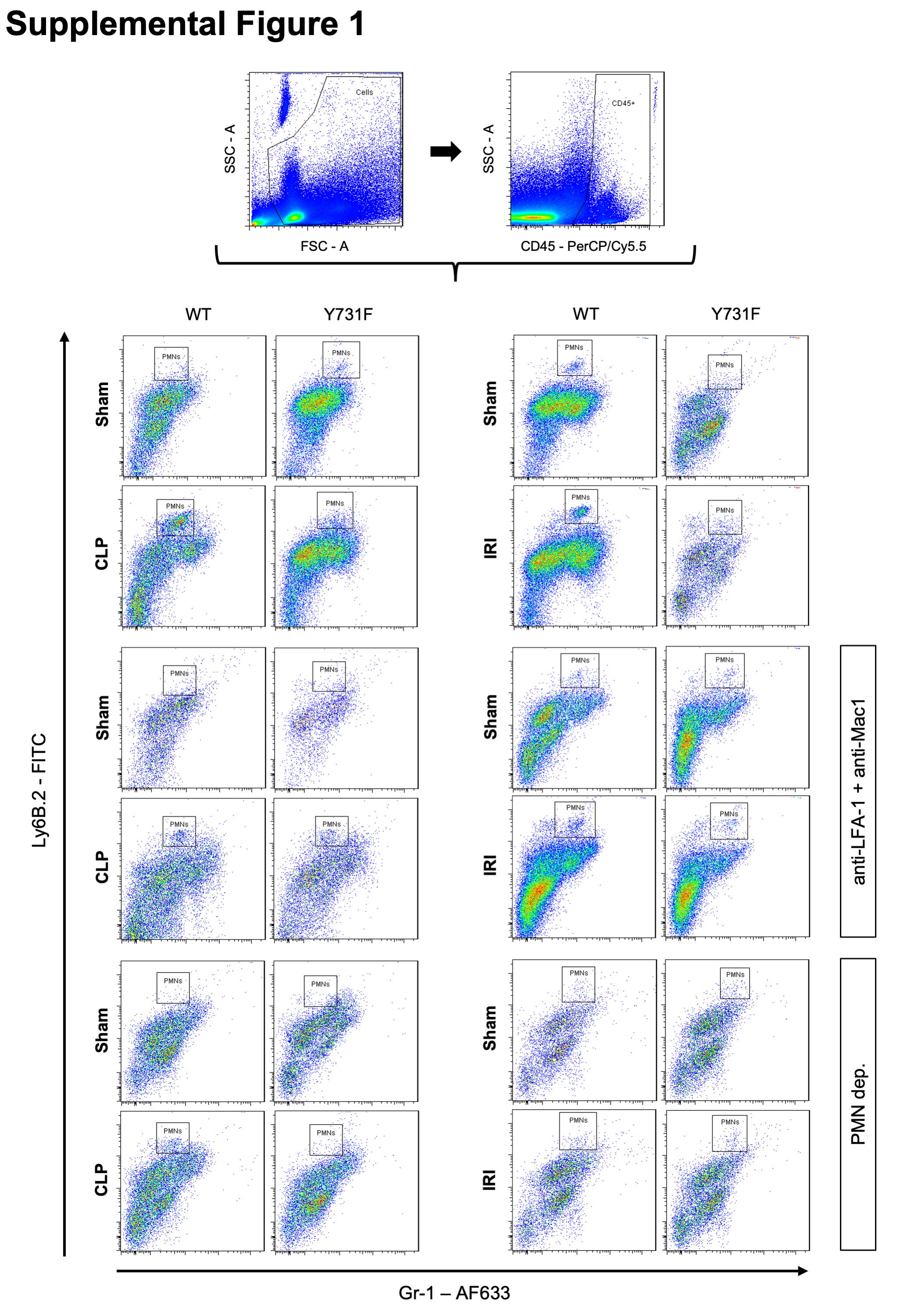

Supplement: Supplementary Figure 1 — Flow cytometry gating strategy for the determination of neutrophil counts after CLP and IRI. AKI was induced in WT and VEC-Y731F mice by CLP or IRI. Sham animals served as appropriate controls. Kidneys were enzymatically digested, and neutrophils were stained with CD45-PerCP/Cy5.5 (clone 30-F11), Gr-1-AF633 (clone 1A8) and Ly6B.2-FITC (clone 7/4). Cells were chosen by FSC-A/SSC-A size discrimination. CD45+-leukocytes were finally examined for a Gr-1+Ly6B.2+ population. The double positive population was considered as neutrophils and total counts were determined for further calculations. [file Image_1.tiff]

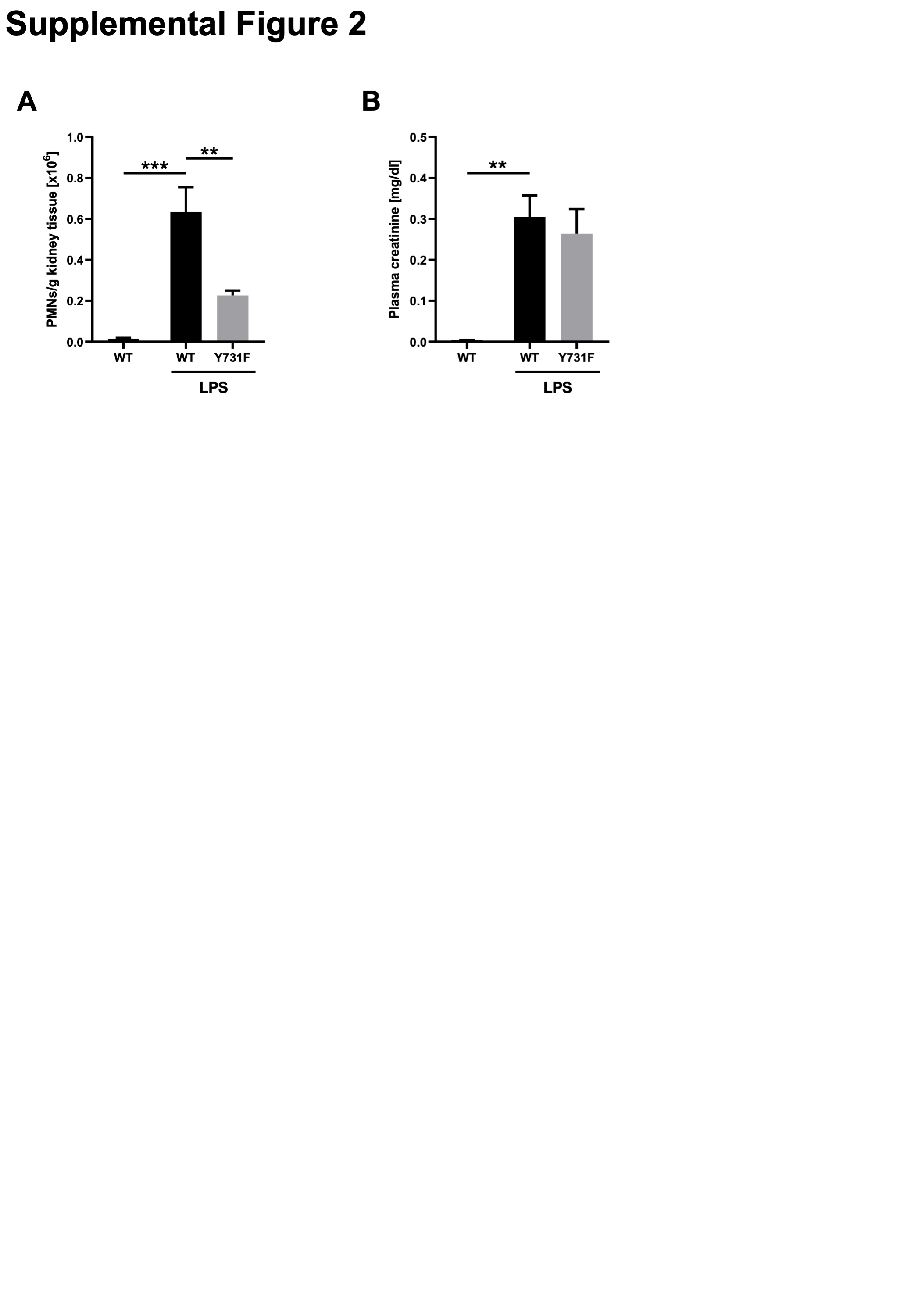

Supplement: Supplementary Figure 2 — LPS-induced sepsis results in AKI independent of neutrophil recruitment. WT and VEC-Y731F mice were injected with 10 µg LPS/ g body weight. Neutrophil recruitment into the kidneys (A) and plasma creatinine (B) were analyzed 24 h after injection. WT mice injected with PBS served as sham controls (n=5). Data are mean ± SEM. ***p < 0.001, **p < 0.01. [file Image_2.tiff]

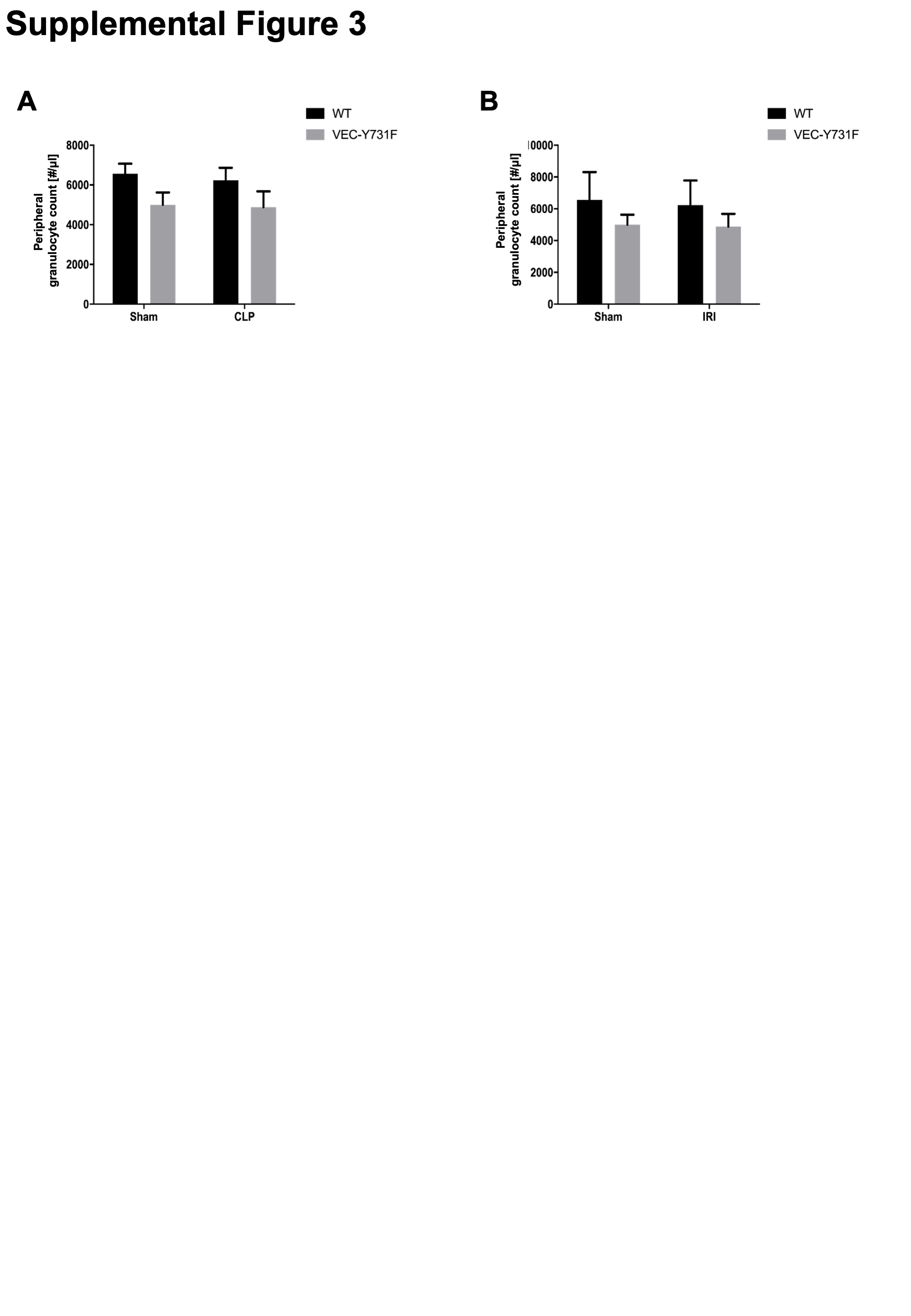

Supplement: Supplementary Figure 3 — Peripheral blood granulocyte counts. Whole blood samples were obtained from WT and VEC-Y731F mice before CLP- (A) or IRI-surgery (B). The number of granulocytes in peripheral blood was analyzed by kimura staining using a modified Neubauer chamber (n=6-10). Data are mean ± SEM. [file Image_3.tiff]

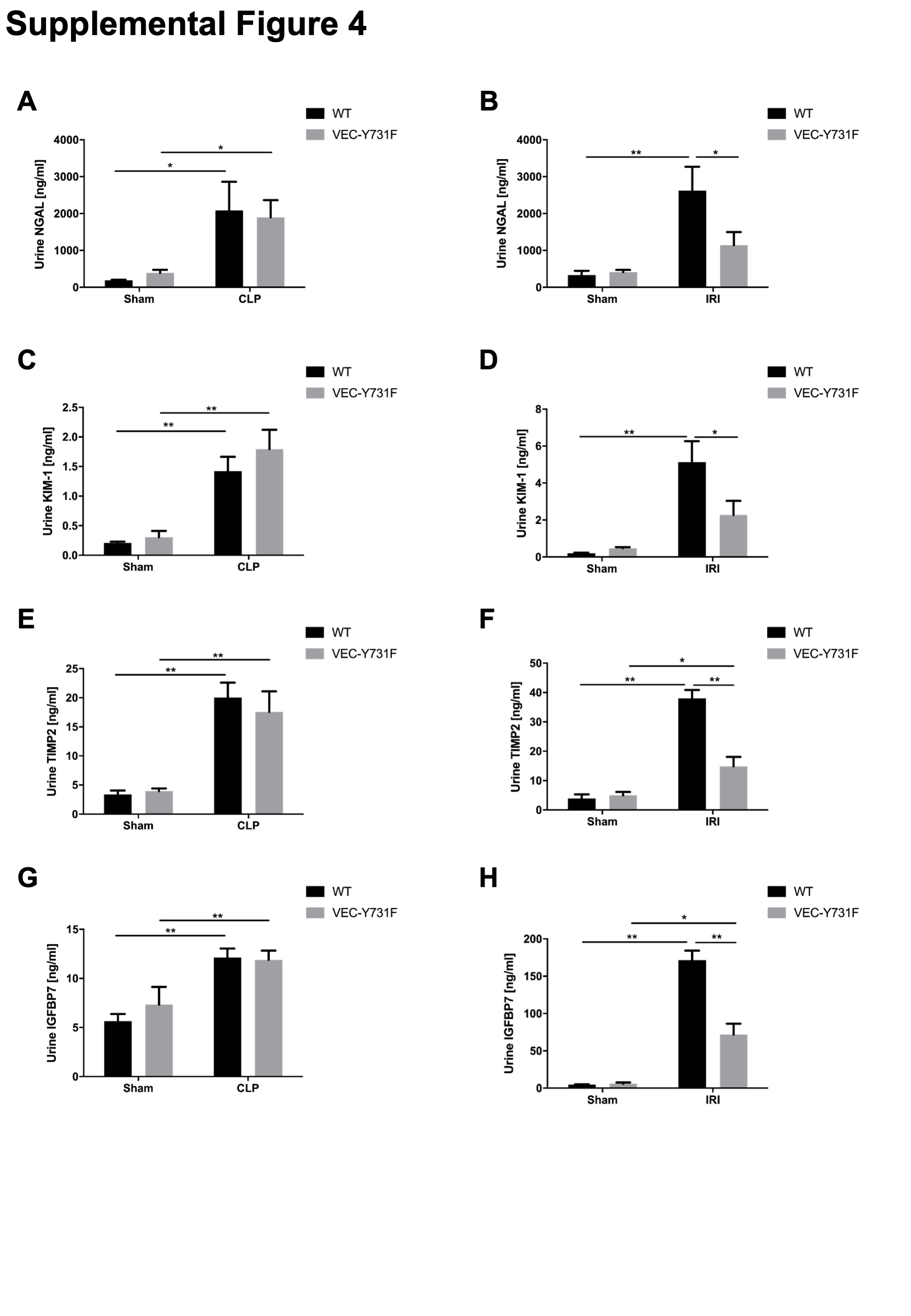

Supplement: Supplementary Figure 4 — Impaired neutrophil recruitment decreases urinary concentration of renal injury biomarkers only in IRI- but not CLP-induced AKI. AKI in WT and VEC-Y731F mice was induced either by CLP or IRI. 24 hours after AKI-induction by CLP- or IRI-surgery the concentrations of the biomarkers NGAL (A, B), KIM-1 (C, D), TIMP2 (E, F) and IGFBP7 (G, H) were measured by ELISA (n=3-8). Data are mean ± SEM. **p < 0.01, *p < 0.05. [file Image_4.tiff]

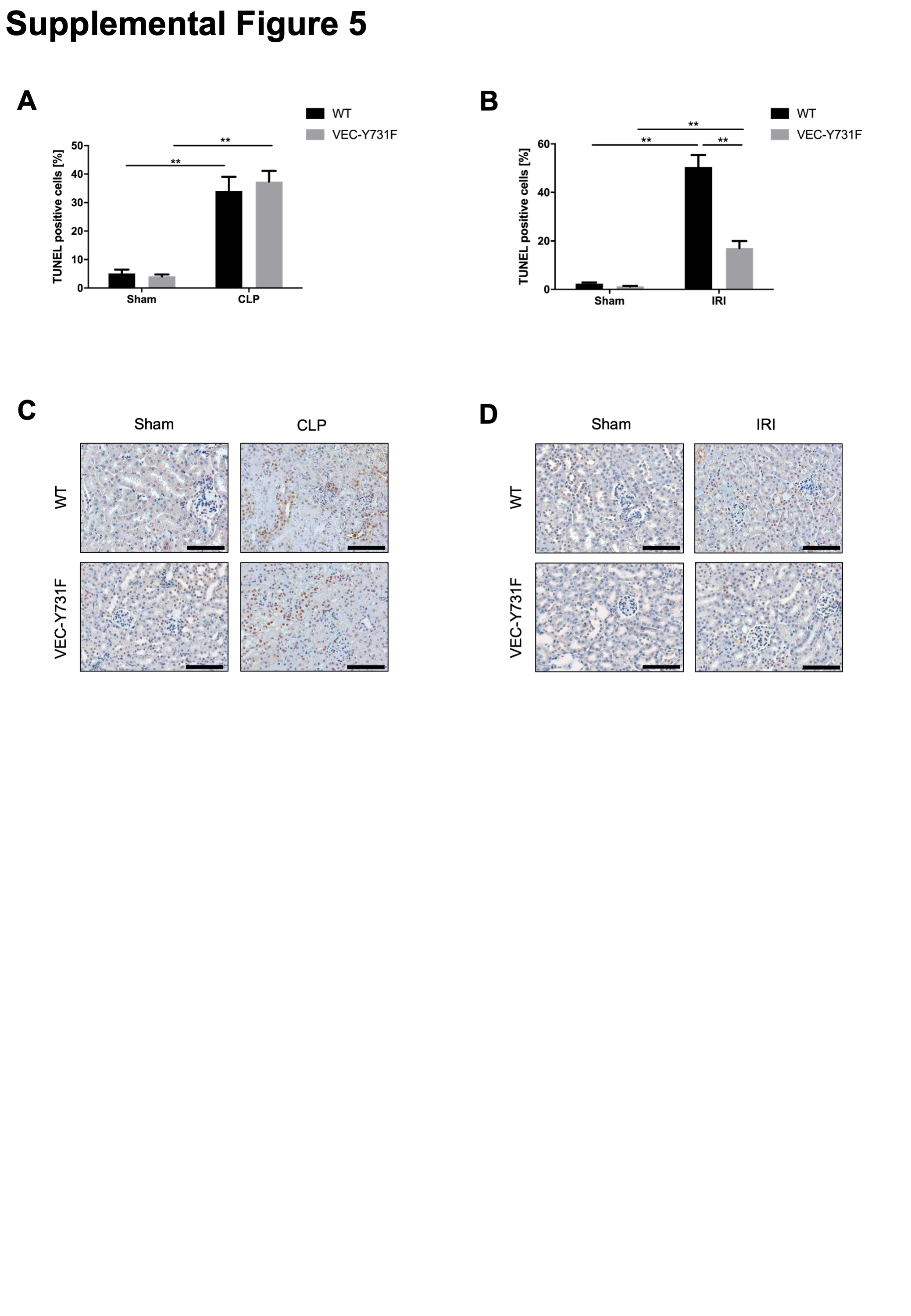

Supplement: Supplementary Figure 5 — Impaired neutrophil recruitment decreases renal tissue apoptosis only in IRI- but not CLP-induced AKI. AKI in WT and VEC-Y731F mice was induced either by CLP or IRI. 24 hours after AKI-induction by CLP- or IRI-surgery renal cortical tissue sections were excised, fixed in formaldehyde, embedded, and processed for TUNEL stainings. The histological analysis was performed from at least 25 high-power fields per kidney from WT and VEC-Y731F mice after CLP- (A) or IRI-surgery (B) and the percentage of TUNEL-positive cells was counted. Exemplary histological images of TUNEL stainings from WT and VEC-Y731F mice after CLP- (C) or IRI-surgery (D) (n=3). Data are mean ± SEM. **p < 0.01. Scale bar: 100 µm. [file Image_5.tiff]

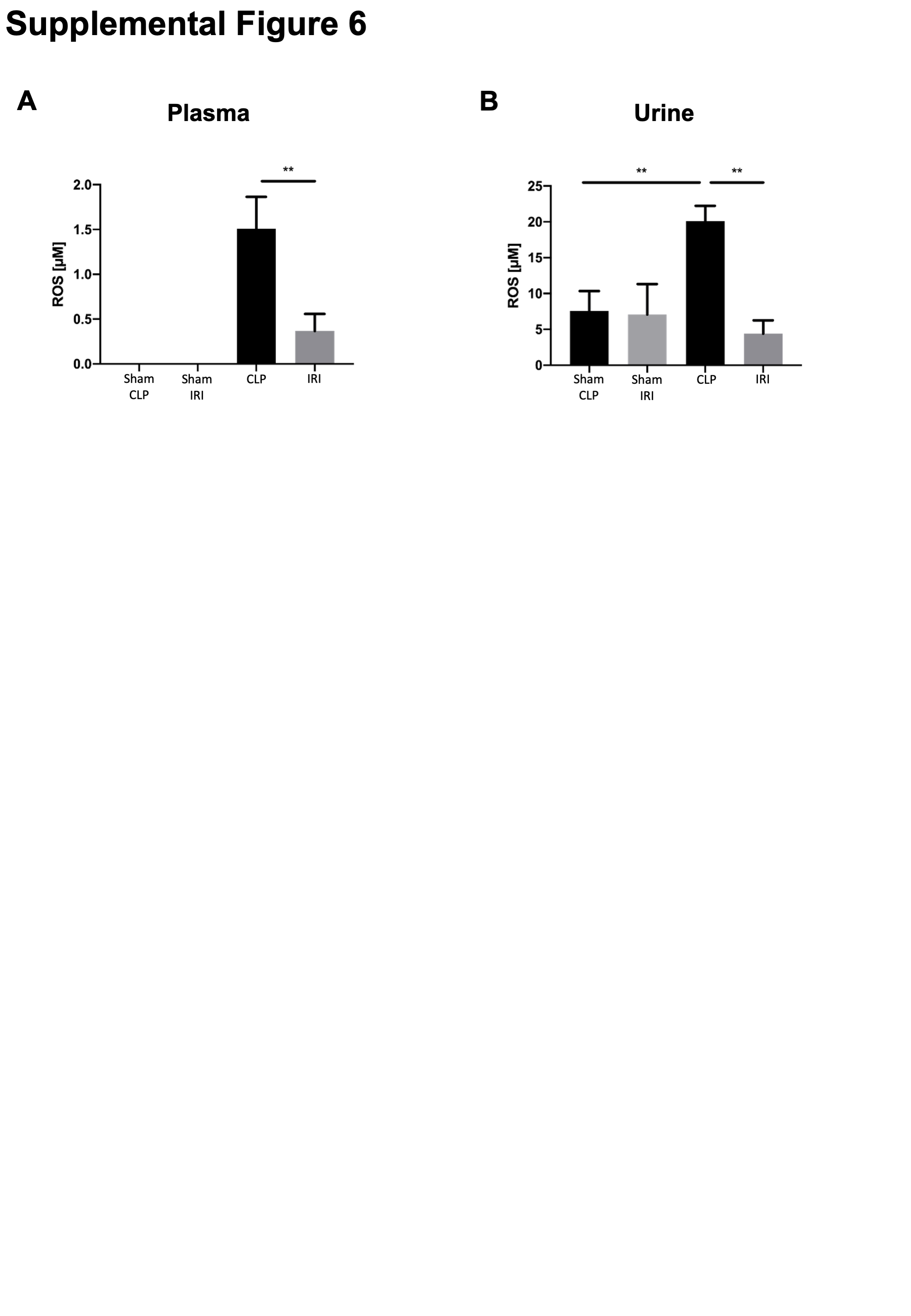

Supplement: Supplementary Figure 6 — ROS levels in plasma and urine after AKI-induction by CLP and IRI. Plasma and urine samples were obtained from WT mice 24 hours after induction of CLP- or IRI-induced AKI. ROS levels in plasma (A) and urine (B) were analyzed by a photometric assay (n=4-5). Data are mean ± SEM. **p < 0.01 [file Image_6.tiff]

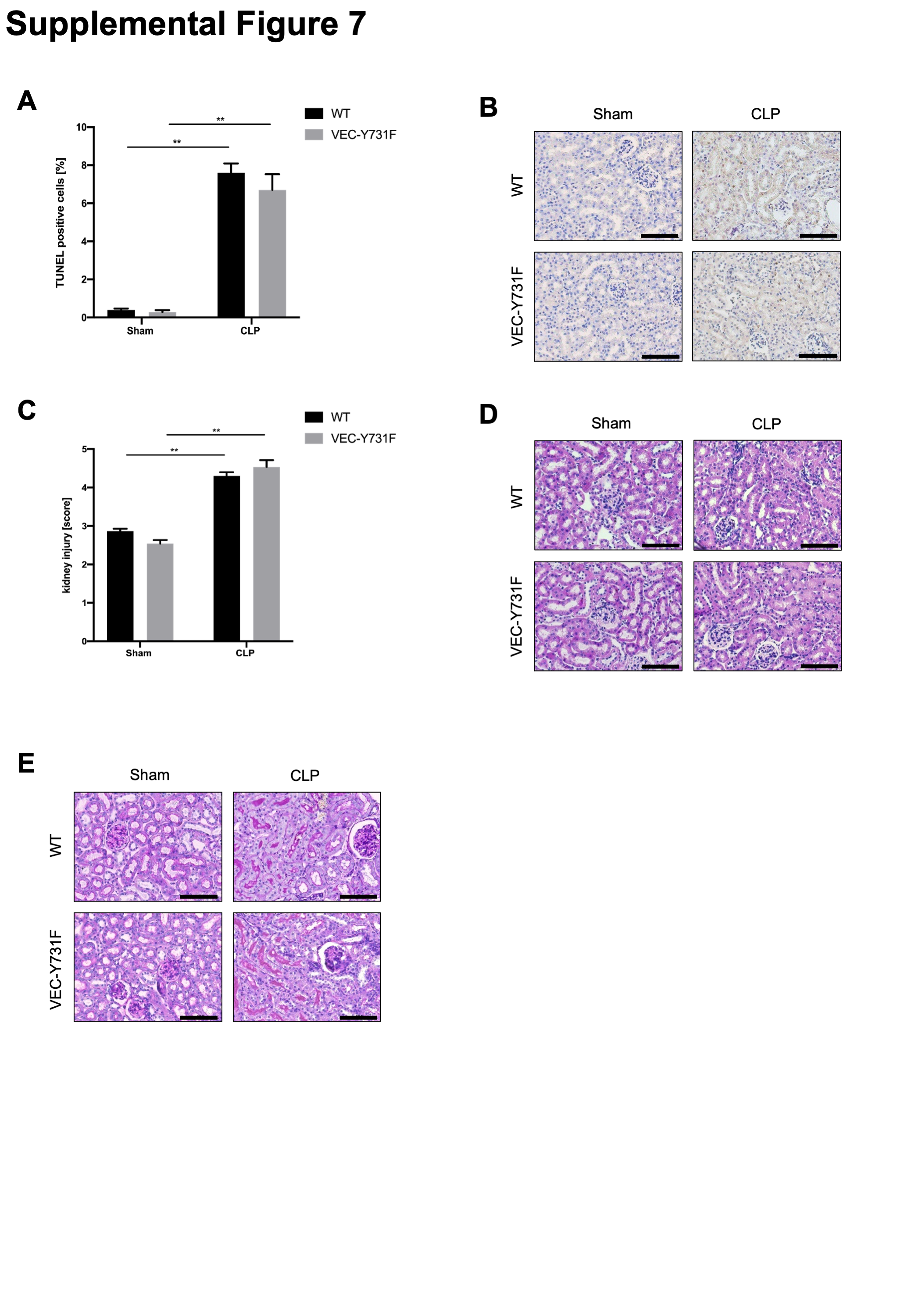

Supplement: Supplementary Figure 7 — The pathogenesis of CLP-induced AKI relies on pro-inflammatory mediators. Sham or CLP-surgery was performed in WT mice. Plasma samples were obtained after 24 hours and 175 µl plasma from sham- or CLP-operated WT mice was injected intravenously into WT and VEC-Y731F mice. After 24 hours renal cortical tissue sections were excised, fixed in formaldehyde, embedded in paraffin, and processed for TUNEL (A, B), H&E (C, D) and PAS (E) stainings. The histological analysis was performed from at least 25 high-power fields per kidney from WT and VEC-Y731F mice after receiving donor plasma from sham- or CLP-operated WT mice. Exemplary images of TUNEL, H&E and PAS stainings (B, D, E) (n=4-5). Data are mean ± SEM. **p < 0.01, *p < 0.05. Scale bar: 100 µm. [file Image_7.tiff]

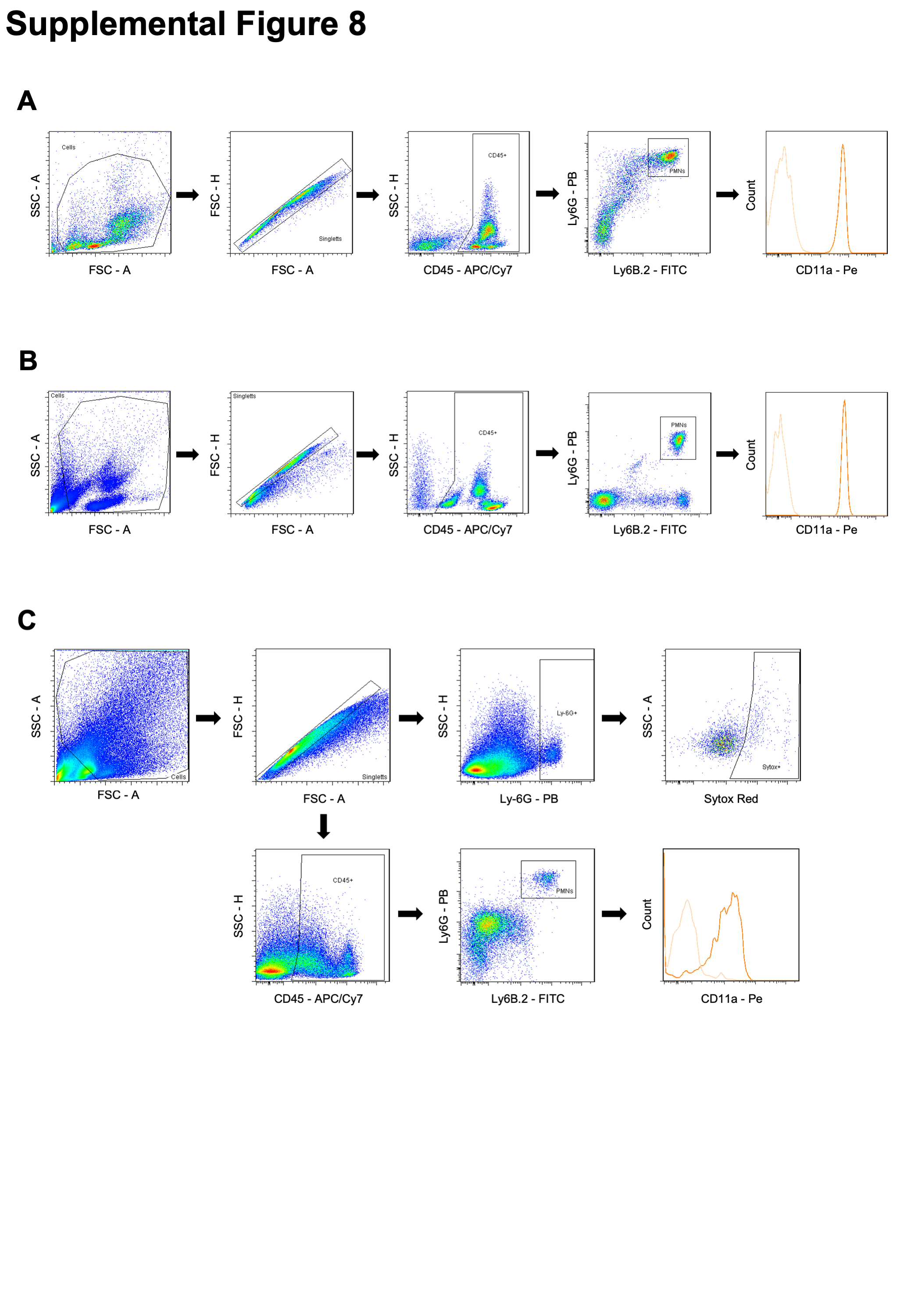

Supplement: Supplementary Figure 8 — Flow cytometry gating strategy for the determination of neutrophil characteristics. Neutrophils were derived from bone marrow and blood of healthy mice. AKI was induced in WT and VEC-Y731F mice by CLP or IRI and kidneys were enzymatically digested. Samples were stained with fluorescent antibodies and analyzed in a FACSCantoII flow cytometer (BD). Cells were chosen by FSC-A/SSC-A and FSC-H/FSC-A size and singlets discrimination, respectively. CD45+Ly-6G+Ly6B.2+ populations were considered as neutrophils and analyzed for Pe signals. Representative gating strategies for bone-marrow-derived (A), peripheral blood-derived (B) and kidney-derived (C) neutrophils. Sytox+ cells were chosen from a Ly-6G+ population. Light orange: isotype control; deep orange: antibody staining. [file Image_8.tiff]
